# Vaccination with a Zika virus envelope domain III protein induces neutralizing antibodies and partial protection against Asian genotype in immunocompetent mice

**DOI:** 10.1186/s41182-022-00485-6

**Published:** 2022-12-05

**Authors:** Minna Shin, Kiju Kim, Hyo-Ji Lee, Yu-Jin Jung, Jeongho Park, Tae-Wook Hahn

**Affiliations:** 1 INNOVAC, Chuncheon, 24341 Republic of Korea; 2 College of Veterinary Medicine & Institute of Veterinary Science, Kangwon National University, Chuncheon, 24341 Republic of Korea; 3 College of Biological Sciences, Kangwon National University, Chuncheon, 24341 Republic of Korea

**Keywords:** Zika virus, Infectious diseases, Disease prevention

## Abstract

**Background:**

Zika virus (ZIKV) is a mosquito-borne flavivirus classified in Flaviviridae family such as dengue (DENV), yellow fever, and West Nile virus. An outbreak of ZIKV infection can pose a major public health risk because the contagion is unpredictable and induces severe pathology such as Guillan-Barre syndrome and neonatal microcephaly. However, an authorized ZIKV vaccine is not yet available, while several vaccine candidates are under development.

**Methods:**

In this study, we constructed a recombinant ZIKV vaccine (Z_EDIII) that includes ZIKV envelope protein domain III using *E. coli* expression system. Then both humoral and cellular immunity were examined in C57BL/6 (female, 8-weeks-old) mice via Indirect ELISA assay, PRNT, ELISpot and cytokine detection for IFN-γ, TNF-α, and IL-12. In addition, the cross protection against DENV was evaluated in pups from Z_EDIII vaccinated and infected dam.

**Results:**

Mice immunized by Z_EDIII produced a significant amount of ZIKV EDIII-specific and neutralizing antibodies. Together with antibodies, effector cytokines, such as IFN-γ, TNF-α, and IL-12 were induced. Moreover, vaccinated females delivered the adaptive immunity to neonates who are protective against ZIKV and DENV challenge.

**Conclusions:**

This study observed Z-EDIII-induced humoral and cellular immunity that protected hosts from both ZIKV and DENV challenges. The result suggests that our ZIKV EDIII recombinant vaccine has potential to provide a new preventive strategy against ZIKV infection.

## Background

Zika virus (ZIKV) is a flavivirus mediated by mosquito bite. Since the first case of ZIKV infection reported in 1947, serious clinical symptoms including Guillan-Barre syndrome and neonatal microcephaly have been reported [1–3]. In the past decade, ZIKV infected more than 1 million people worldwide, with Brazil among the hardest-hit countries, but neither authorized vaccines nor medicines have been available [4, 5]. Therefore, it is critical to develop a strategy for a safe and effective ZIKV vaccine. ZIKV is genetically correlated with other flaviviruses such as Dengue virus (DENV), West Nile virus (WNV), and yellow fever virus (YFV) [6–8]. ZIKV envelope (E) protein consists of three ectodomain structures (EDI, EDII, and EDIII) [4, 9]. The E protein facilitates viral invasion by activating receptor binding, cellular attachment, viral entry, and fusion [10–12]. Neutralizing antibody production is an essential procedure for ZIKV protection, and its significance has been shown in other viruses, such as YFV and Tick-borne encephalitis virus (TBEV) [13–15]. The EDIII-inducing neutralizing antibody has a cellular receptor-binding motif that is critical for overall host antibody response and ZIKV-specific immunity [13, 16–18]. Therefore, a vaccine that borrows the EDIII structure can facilitate protective immunity.

A number of ZIKV vaccines are under development in various forms, including purified-inactivated, live-attenuated, DNA, mRNA, and recombinant vaccines [19]. The recombinant subunit vaccines are based on the ZIKV protein by the expression host system that utilizes bacteria [16, 20], yeast [21], mammalian cells [22], insect cells [23], and transgenic plants [24]. Among these, *E. coli* expression system is the most frequently used because *E. coli* grows fast and has a high protein yield [25]. In this study, we developed a ZIKV recombinant subunit vaccine (Z_EDIII) that carries *E. coli*-expressed EDIII protein and evaluated the immune responses by Z_EDIII. Z_EDIII-immunized mice produced a sizable amount of ZIKV-specific antibody and effector cytokines such as IFN-γ, IL-12, and TNF-α. Neonates from Z_EDIII-immunized females were protected against ZIKV and Dengue virus (DENV) challenge. Therefore, we suggest that Z_EDIII is a potential ZIKV vaccine candidate.

## Methods

### Cells and viruses

Vero cells (KCLB, Seoul, Korea) were grown in Minimum Essential Medium (*MEM)-α* supplemented with 10% fetal bovine serum (FBS; Gibco, Grand Island, NY, USA), and Vero 76 cells (ATCC, Manassas, VA, USA) were grown in Dulbecco’s Minimal Essential Medium (DMEM; Gibco, Carlsbad, CA, USA) supplemented with 10% FBS at 37 °C in 5% CO_2_ until monolayers were observed. We used two ZIKV strains, PRVABC59 and MR766 which were propagated in Vero and Vero 76 cells respectively at a multiplicity of infection of 0.01. ZIKV stocks were titrated by plaque assay using two cell lines and stored at − 80 °C.

### Plasmids construction and protein expression

The recombinant BL21 star (DE3) was commercially constructed (GenScript, Piscataway, NJ, USA) using the *E. coli* expression system. Briefly, the ZIKV EDIII protein of strain ZBRX6 (amino acid 592–745, Genbank Acc.No. AQS26809.1) was synthesized with optimized *E. coli* codons (GenScript, Piscataway, NJ, USA). Recombinant BL21 star (DE3) was inoculated into TB medium containing related antibiotic and cultured at 37 ℃. When the OD_600_ reached about 1.2, cell culture was induced with IPTG at 37 ℃ for 4 h. Cells were harvested by centrifugation. Cell pellets were resuspended with lysis buffer followed by sonication. The precipitate after centrifugation was dissolved using a denaturing agent. Z_EDIII was obtained by two-step purification using an Ni column and a Superdex 75 column. Z_EDIII was sterilized using a 0.22 μm filter before being stored in aliquots. The protein purity and molecular weight were determined by sodium dodecyl sulfate–polyacrylamide gel electrophoresis (SDS-PAGE) and Western blot analysis. The primary antibody for Western blot was mouse-anti-His mAb (GenScript, Piscataway, NJ, USA). The protein concentration was determined using a Bradford protein assay with bovine serum albumin (BSA) standard curve.

### Mice and immunization

C57BL/6 (female, 8-weeks-old) mice were purchased from Orient Bio Inc (Gyeonggi-do, Korea). The mice were housed in a specific pathogen-free facility (the Animal Laboratory Center of Kangwon National University) under a 12-h light–dark cycle and given free access to food and water. Two groups of 8-week-old female C57BL/6 mice (*n* = 5 in each group) were vaccinated with 10 μg recombinant ZIKV EDIII protein (Z_EDIII) or phosphate buffered saline (PBS). The vaccine was mixed with 500 μg of aluminum hydroxide gel (Invivogen, San Diego, CA, USA) and 20 μg of monophosphoryl Lipid A (MPLA; Sigma‐Aldrich, St. Louis, MO, USA). The vaccine–adjuvant mixture was administered via the intramuscular route at 0, 14, and 42 days. At 7, 17 and 45 days post-immunization (dpi), Some groups mice were euthanized with CO_2_ and collected splenocytes for examination of cellular immune response. Mouse sera were collected at 0, 14, and 49 dpi and immunized females were mated with homozygous males. One-day-old neonates were inoculated subcutaneously with each ZIKV strain (MR766, PRVABC59) or PBS as a negative control. Mice were inoculated with 10^6.8^ tissue-culture-infected doses (TCID)_50_/mouse for the MR766 strain and 10^6.3^ TCID_50_/mouse for the PRVABC59 ZIKV strain and were monitored daily until 21 dpi to assess survival rate and weight loss. Serum was collected at 0 and 2 dpi. Mice were euthanized with CO_2_ at the end of the experiment. For this study, no animals were euthanized for sickness or distress. This work was carried out in compliance with the ARRIVE guidelines and approved by the Institutional Animal Care and Use Committee of Kangwon National University (Nos. KW-190131-1, KW-190515-2). All methods were carried out in accordance with relevant guidelines and regulations.

### Enzyme-linked immunosorbent assay (ELISA)

The ZIKV E protein-specific antibodies in sera were detected using an ELISA kit (Alpha Diagnostic, USA) according to the manufacturer’s instructions. Briefly, 100 μl aliquots of diluted serum (1:50,000) were added per well. Z_EDIII-specific antibodies (IgG, IgG1, and IgG2c) in sera were determined by indirect ELISA as described previously [26, 27]. Microplates (Nunc-Immuno Plates; Thermo Scientific, UK) were coated with Z_EDIII (1 μg/ml) at 4 °C overnight. Each well was washed with 0.05% Tween 20 in PBS (PBST) and then with 1% BSA in PBS and incubated at 37 °C for 2 h. The plates were washed and then diluted serum was incubated at 37 °C for 2 h. Plates were washed and incubated with horseradish peroxidase-conjugated goat anti-mouse IgG heavy and light chain antibody (1:10,000; Bethyl Laboratories, TX, USA), goat anti-mouse IgG1 (1:10,000; Bethyl Laboratories), or goat anti-mouse IgG2c (1:8000; Southern Biotech, USA) at 37 °C for 1 h and washed with PBST. Color development was performed using tetramethylbenzidine substrate (Surmodics, USA) and stopped with 2 N H_2_SO_4_. The optical density of the plates was read at 450 nm in an ELISA plate reader (BioTek, Winooski, NT, USA).

### Plaque reduction neutralization test

A plaque reduction neutralization test (PRNT) was performed as described previously [27]. Vero or Vero 76 cells (1 × 10^5^ cells/well) were seeded in 24-well plates and cultured overnight in DMEM medium containing 10% FBS. Serum samples were heat inactivated at 56 °C for 30 min and serially diluted in DMEM. ZIKV and DENV were diluted to 2 × 10^2^ plaque-forming units (PFU)/ml in DMEM, mixed with an equal volume of diluted serum and incubated at 37 °C for 30 min. Then, Vero or Vero 76 cells were incubated with the serum mixture at 37 °C for 2 h. Cells were then washed and cultured at 37 °C for 5 or 14 days in DMEM containing 1% sea plaque agar or 1.4% methyl cellulose. Cells were fixed in 4% paraformaldehyde and stained with 0.1% crystal violet dye. The percentage inhibition of virus infectivity was calculated by counting the number of plaques in immune sera.

### Measurement of cytokine and T cell phenotype

The IFN-γ-secreting splenocytes were measured at 7, 17, and 45 dpi using a mouse IFN-γ enzyme-linked immune absorbent spot (ELISpot) assay (BD Life Sciences, USA). Briefly, 5 × 10^5^ splenocytes/well were stimulated with Z_EDIII (1 μg/ml). Spots were counted under a dissecting microscope (Olympus, model no. SZH-ILLB). To measure secreted cytokines, splenocytes (5 × 10^5^/well) were stimulated with Z_EDIII (1 μg/ml) for 48 h and the supernatant of splenocytes was collected. The levels of cytokines were determined using mouse tumor necrosis factor (TNF)-α ELISA MAX™ standard set, mouse IFN-γ ELISA MAX™ standard set, and mouse interleukin (IL)-12 ELISA MAX™ standard set (BioLegend, USA). Each assay was performed in triplicate. To analyze T lymphocyte subtypes, splenocytes were stained with the following antibodies at a dilution of 1:200 with 1% BSA in PBS at 4 °C for 1 h: anti-mouse CD3 (clone 145-2C11, BD Biosciences), and anti-mouse CD4 (clone RM4-5, BD Biosciences) or anti-mouse CD8 (clone 53–6.7, BD Biosciences) as described previously [27]. Cell phenotype was analyzed using a FACS Calibur flow cytometer (Becton Dickinson).

### Tissue culture infective dose_50_ (TCID_50_)

The TCID_50_ assay was performed as described previously [27]. Vero or Vero 76 cells were seeded in 96-well plates (2 × 10^4^ cells/well) and cultured overnight in DMEM medium containing 10% FBS. Serum samples were heat inactivated at 56 °C for 30 min and serially diluted (10^1^–10^3^) in PBS. The serum samples were used to infect Vero or Vero 76 cells at 37 °C for 1 h. Cells were then washed and cultured at 37 °C for 14 days in complete DMEM. TCID_50_ was calculated using the end-point method, and virus infectivity was determined using the Karber method.

### Statistical analysis

Statistical analysis was performed using GraphPad Prism (v. 5.0; GraphPad Software, La Jolla, CA, USA) using one-way analysis of variance with Tukey’s multiple comparisons test to compare groups. *P* values < 0.05 were considered significant.

## Results

### Expression of recombinant ZIKV envelope protein domain III

**Fig. 1:**
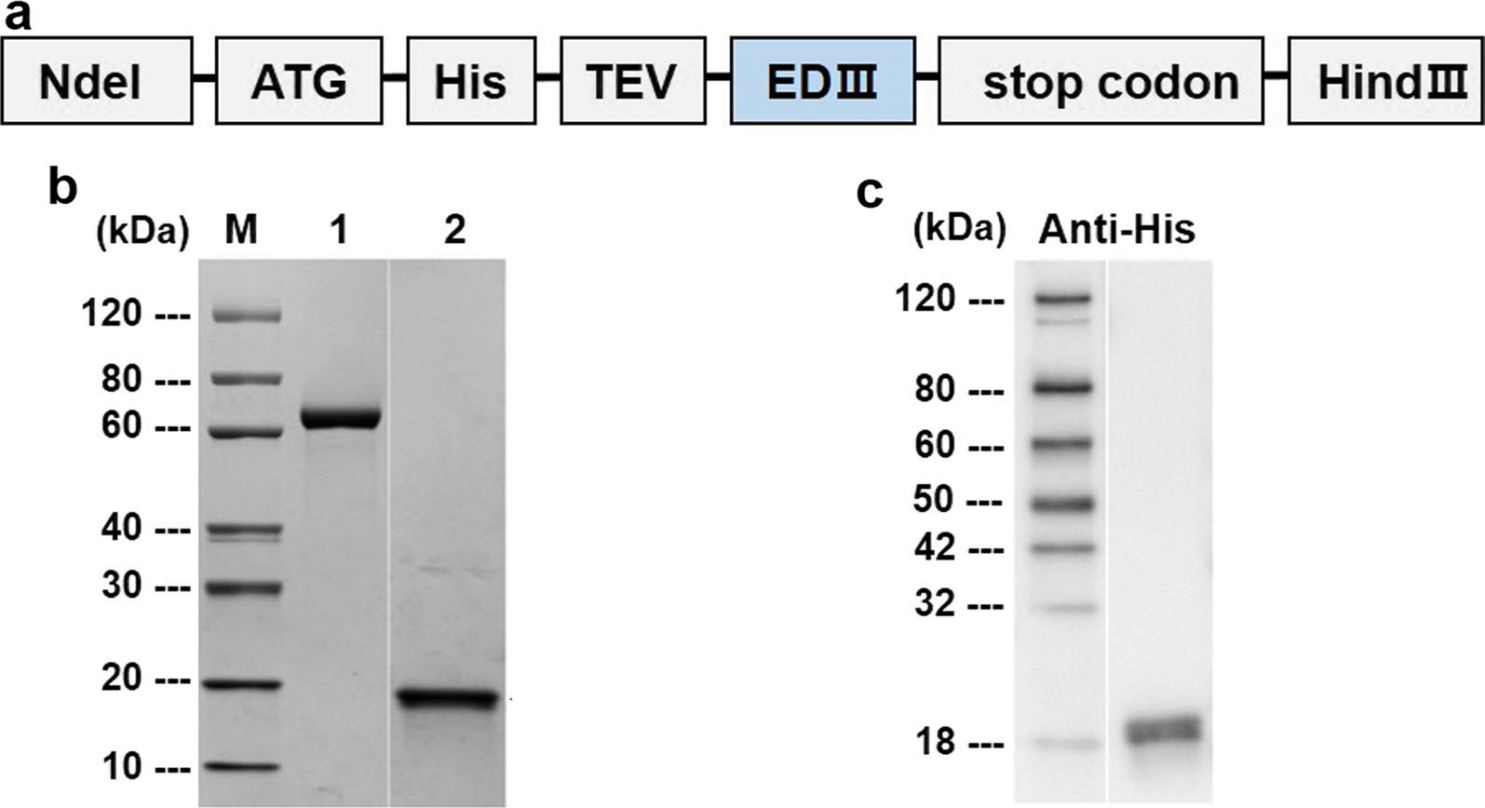
Construction of Z_EDIII. **a** Schematic of constructs expressing Z_EDIII (TEV: TEV protease site). **b**, **c** Purified Z_EDIII proteins were confirmed by SDS-PAGE **b** and Western blot **c**. **b** Lane M: Protein Marker (GenScript, Cat. No. M00516). Lane 1: BSA (2 μg). Lane 2: Target protein (Z_EDIII 2 μg). **c** The primary antibodies used anti-His antibody (Mouse-anti-His mAb, GenScript, Cat. No. A00186)

Previous studies have shown that EDIII is a major target of ZIKV-specific antibodies [13, 16] and we constructed a recombinant subunit vaccine that includes the EDIII protein. The coding sequence of the EDIII protein (amino acid 592–745, Genbank Acc.No. AQS26809.1) in a Brazilian ZIKV strain (ZBRX6) was inserted into an expression vector (pET28a) and cloned in *E. coli* cells (Fig. 1a). Viral protein was purified using an Ni column and a Superdex75 column and the expression of EDIII (18 kDa) protein was confirmed by SDS-PAGE and Western blotting (Fig. 1b and c). The recombinant vaccine is termed Z_EDIII.

### Z_EDIII-induced humoral immune response

**Fig. 2:**
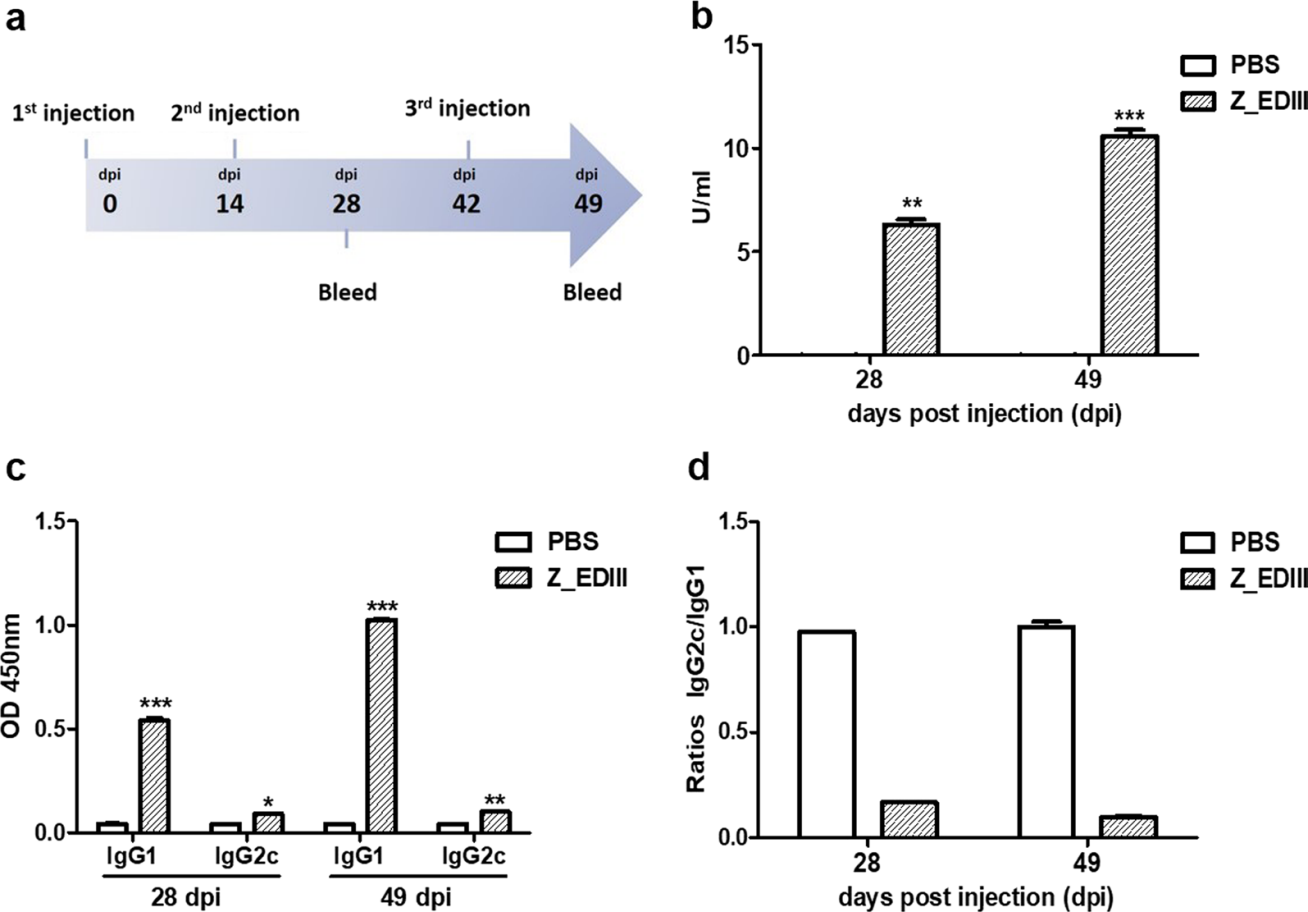
ZIKV envelope protein domain III-specific antibody responses elicited by Z_EDIII. **a** Experimental strategies. C57BL/6 mice (*n* = 5 per group) were immunized with Z_EDIII or PBS at 0, 14, and 42 dpi. For Ag-specific IgG antibodies, blood samples were collected at 0, 28, and 49 dpi. **b** Serum samples were diluted (1:50,000) and OD 450 values were measured using ZIKV envelope protein domain III-coated ELISA kit. The ZIKV envelope protein domain III-specific IgG antibody activity units were calculated. **c** Serum samples were diluted (1:100) and OD 450 values were measured. **d** IgG2c-to-IgG1 ratio in Z_EDIII- or PBS-immunized mice is shown. Data show the mean ± SD of five mice per group. Each sample was assayed in duplicate. Statistical analysis *p < 0.05, **p < 0.01, ***p < 0.001

During ZIKV infection, IgG for envelope glycoprotein (E) is produced [6]. The major target of human monoclonal antibodies is EDIII, but other transmembrane proteins (EDI and EDII) are also covered by antibodies [28–33]. Moreover, previous studies suggest that EDIII provides epitopes that are targeted by both B cell repertoire and multiple fractions in polyclonal immune sera [8, 29, 31, 33, 34]. To examine whether our Z_EDIII protein induces ZIKV envelope protein domain III-specific IgG antibodies, we performed an ELISA assay. Compared with PBS-injected control, Z_EDIII immunization significantly increased Z_EDIII-specific IgG in immunocompetent mice 4 weeks after immunization (Fig. 2a and b). Z_EDIII immunization in immunocompetent mice 7-weeks after immunization boosted the pathogen-specific antibody production by 1.7 times. This indicates that our Z_EDIII immunization induces sufficient amount of Ag-specific antibodies.

By Ag-specific detection and a plaque reduction neutralization test, we evaluated the neutralizing capacity by Z_EDIII immunization against two different ZIKV strains: MR766 and PRVABC59. The neutralizing antibody was detected from serum in 7-week-old mice after Z_EDIII immunization but not from PBS-injected control (Table 1). We further analyzed Ag-specific IgG1 and IgG2c from Z_EDIII immunized mice and evaluated the type of immune responses. Both IgG subtypes were promoted by Z_EDIII immunization; the level of IgG2 was lower than that of IgG1 (Fig. 2c and d). Accordingly, we speculate that Z_EDIII is a good immune stimulator, and the administration is more likely to induce Th2 than Th1 response.

**Table 1:** Level of neutralizing antibodies against two ZIKV strains and a dengue virus (DENV-2) strain in immunized mice

Injected group	Titer (PRNT_50_)		
	ZIKV (MR766)	ZIKV (PRVABC59)	DENV-2
PBS	0	0	0
Z_EDIII	20	20	20

### Z_EDIII-induced cellular immune response

**Fig. 3:**
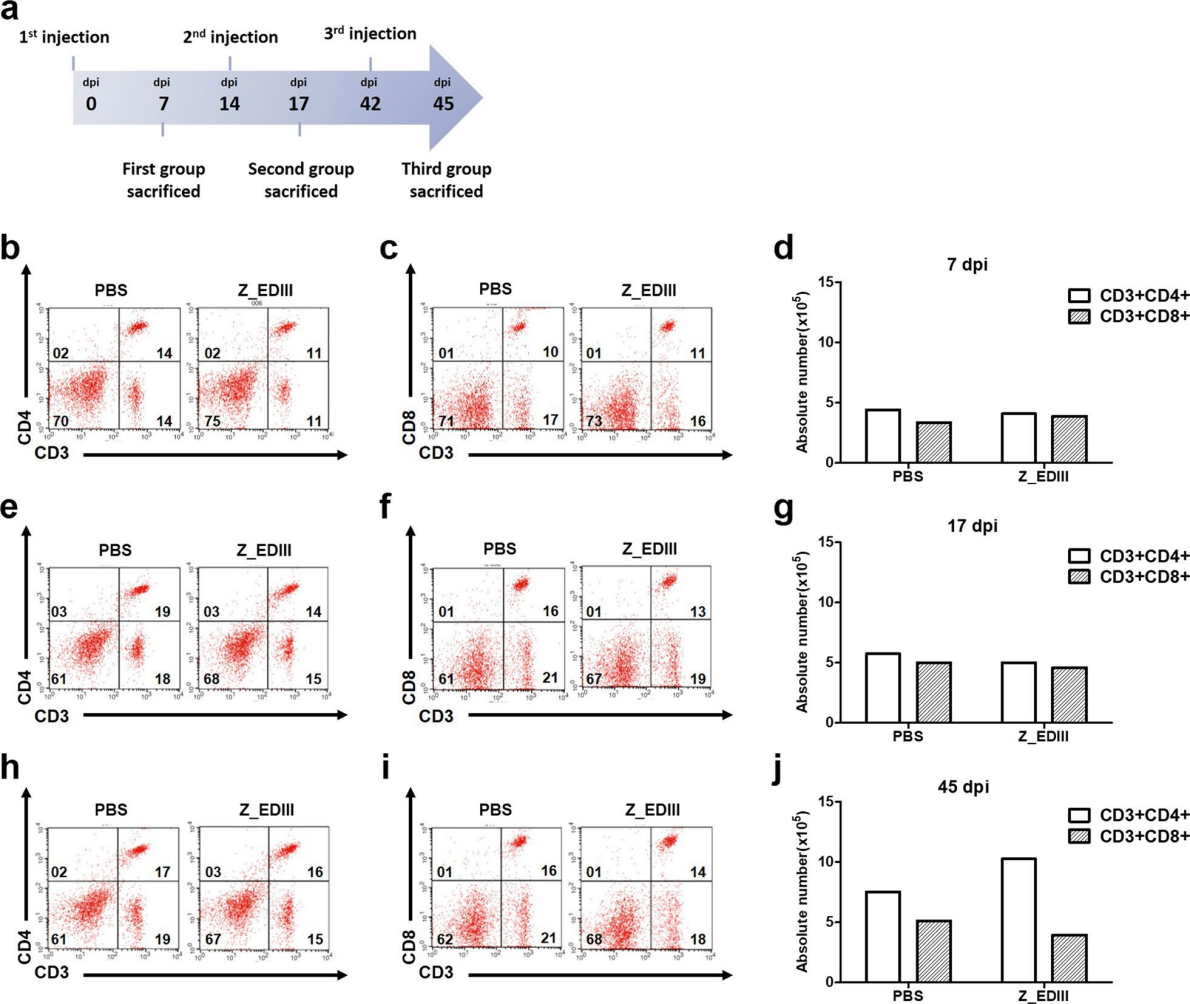
CD4^+^ and CD8^+^ T cells population in immunized mice. **a** Experimental procedure for cellular immune response. C57BL/6 mice (*n* = 5) were immunized with Z_EDIII or PBS at 0, 14, and 42 dpi. Mice were euthanized at 7, 17, and 45 dpi (7 dpi; **b**–**d**, 17 dpi; **e**–**g**, 45 dpi; **h**–**j**). Total splenocytes were collected from five mice in each group and stained with antibodies to CD3, CD4, and CD8 molecule

**Fig. 4:**
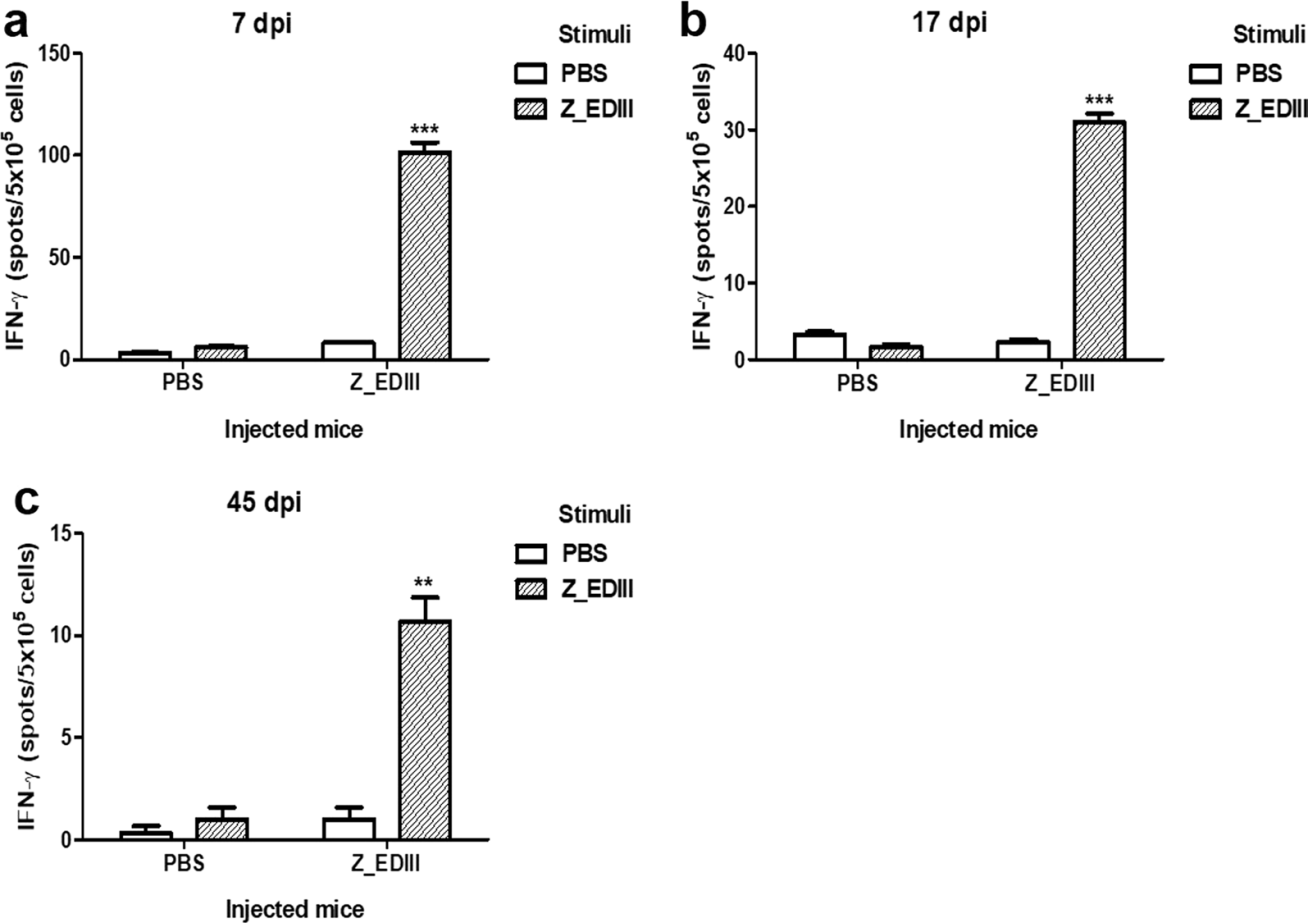
Number of IFN-γ-secreting splenocytes in immunized mice. C57BL/6 mice (*n* = 5) were immunized with Z_EDIII or PBS at 0, 14, and 42 dpi. Mice were euthanized 7, 17, and 45 dpi (7 dpi; **a**, 17 dpi; **b**, 45 dpi; **c**). Splenocytes were stimulated with Z_EDIII (10 μg/ml) or PBS. Ag-specific IFN-γ response was identified by ELISPOT assay at 48 h after stimulation. Data show mean ± SD of five mice per group. Each sample was assayed in triplicate. Splenocytes were pooled from five mice in each group. Statistical analysis *p < 0.05, **p < 0.01, ***p < 0.001

To evaluate vaccination-induced cellular immunity, we examined the effect of Z_EDIII doses on T cell phenotype (Fig. 3a). Total splenocytes were stained with antibodies to CD4 and CD8 molecules. Single and double doses of Z_EDIII did not change the frequencies of CD4+ and CD8+ T cells (Fig. 3b–g). However, triple immunizations increased the CD4+ T cell but slightly suppressed the CD8+ T cell population. Additionally, more CD4+ T cells were observed than CD8+ T cells in the spleen after triple immunizations (Fig. 3h–j). Next, we evaluated cellular immunity function by examining effector cytokines from immunized animals. IFN-γ and TNF-α are pivotal cytokines that are produced by CD4+ and CD8+ T cells after ZIKV antigen stimulation [35]. When we quantified IFN-γ-secreting cells by ELISpot assay, a single dose increased cytokine production by 12 times (Fig. 4a). Double and triple doses also promoted IFN-γ-producing cells but not by as many as a single dose (Fig. 4b, c).

**Fig. 5:**
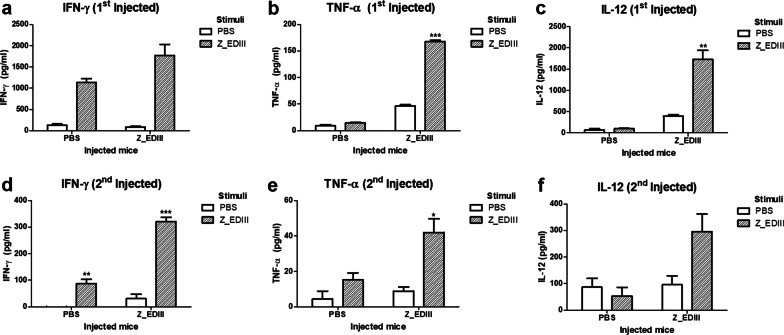
Cytokine production elicited by Z_EDIII immunization. C57BL/6 mice (*n* = 5) were immunized with Z_EDIII or PBS at 0 and 14 dpi. Mice were euthanized at 7 and 17 dpi (7 dpi; **a**–**c**, 17 dpi; **d**–**f**). Splenocytes were pooled from five mice in each group and stimulated with Z_EDIII (10 μg/ml) or PBS for 48 h. Concentrations of IFN-γ, TNF-α and IL-12 from the supernatant by ELISA. Data show the mean ± SD of five mice per group. Each sample was assayed in triplicates. Statistical analysis *p < 0.05, **p < 0.01, ***p < 0.001

Effector cytokine production by Z_EDIII was also investigated by ELISA assay. Levels of IFN-γ and TNF-α were identified and we additionally examined IL-12 level, which is originated antigen presenting cells and activates Th1 differentiation [36, 37]. Like the ELISpot assay, splenocytes from single-dosed mice secreted a high level of cytokines when cells were restimulated with ZIKV antigen (Fig. 5a–c). Double doses also promoted cytokine secretion, but the amount was lower compared with that induced by a single dose (Fig. 5d–f). On the other hand, triple doses completely blocked cytokine secretion (data not shown). The results suggest that Z_EDIII immunization is an effective inducer for effector cytokines, though multiple administrations make immune cells exhausted.

### Immunization with Z_EDIII protects against ZIKV or DENV challenge in immunocompetent neonatal mice

**Fig. 6:**
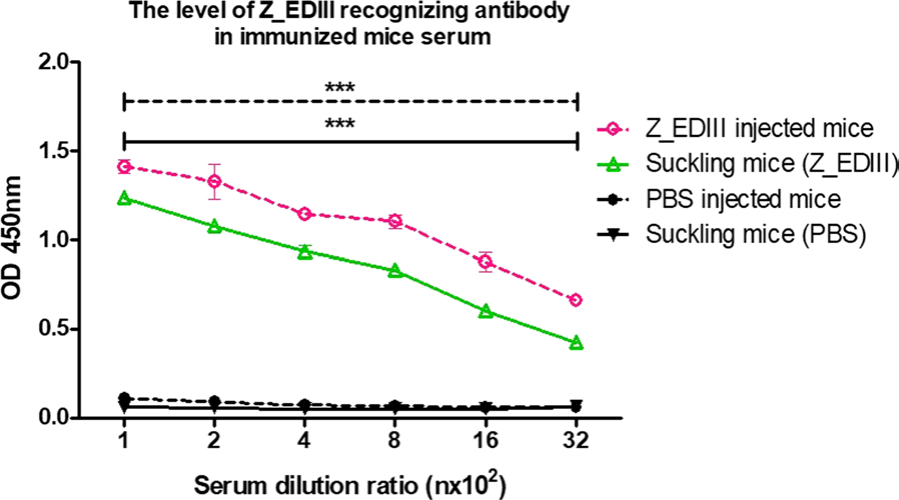
The level of Z-EDIII specific antibody after immunization. C57BL/6 mice (*n* = 5 per group) were immunized with Z_EDIII or PBS at 0, 14, and 42 dpi. Immunized mice were mated with homozygous breeding pairs. To measure Z_EDIII-specific IgG antibodies, blood samples were collected at day 2. Serum samples were serially diluted from 1:100 to 1:3,200 and the antibody level was determined by ELISA. Data show the mean ± SD of five mice per group. Each sample was assayed in duplicate. Statistical analysis *p < 0.05, **p < 0.01, ***p < 0.001

Controlling ZIKV spread during gestation is critical because the viral transmission across placenta induces high risk of intrauterine growth restriction (IUGR), spontaneous miscarriage, and microcephaly [38]. Accordingly, the impact of trans-generational immune priming by Z_EDIII was evaluated. Circulating Z_EDIII-specific IgG level was measured in neonates from triple-dosed parents. As a result, the pups showed a significantly increased level of Ag-specific IgG compared with the PBS-administered group (Fig. 6).

**Fig. 7:**
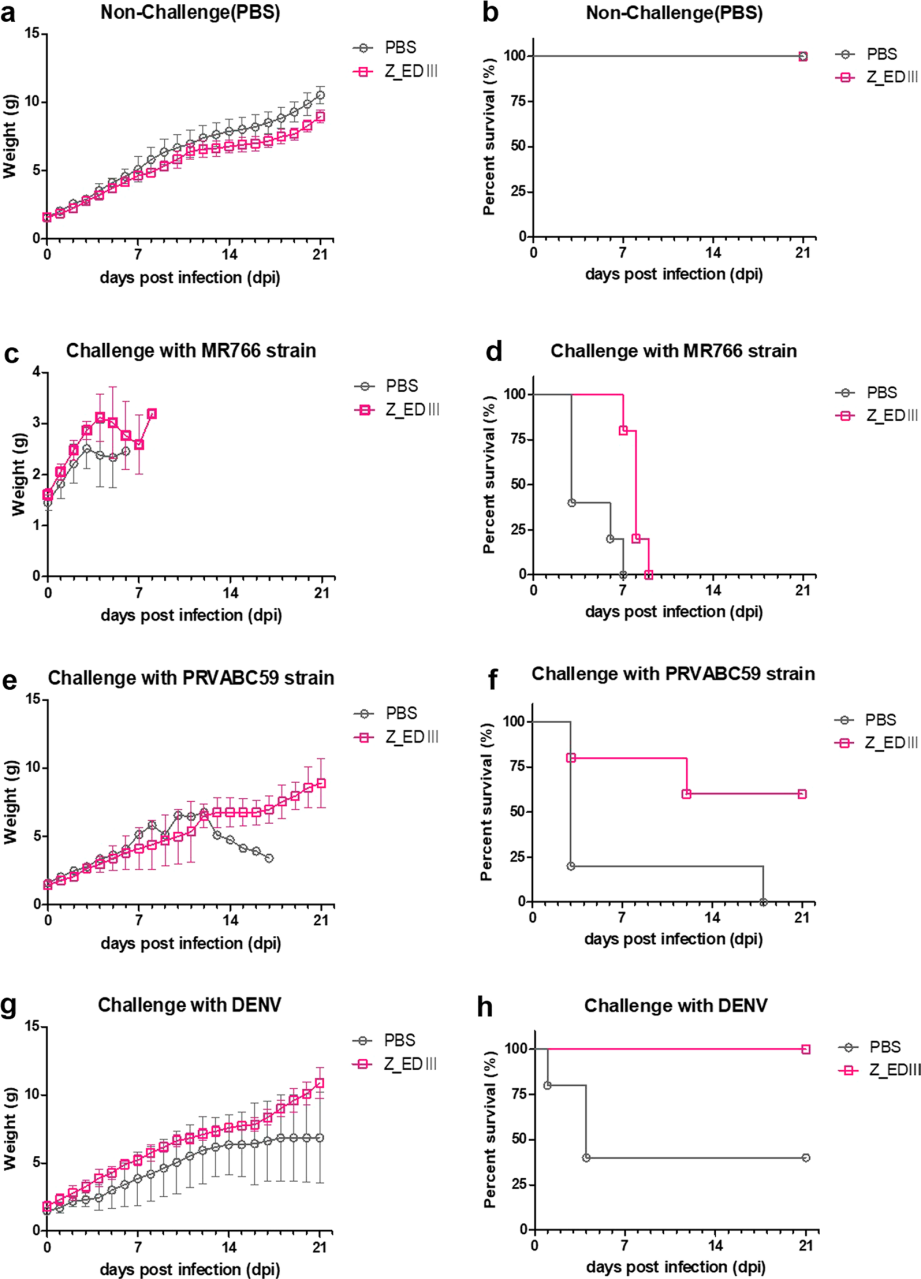
Clinical results in neonates against ZIKV and DENV challenge. Body weight changes and survival rates were observed. C57BL/6 mice (*n* = 5 per group) were immunized with Z_EDIII or PBS at 0, 14, and 42 dpi. Immunized females were mated with homozygous breeding pairs. The survival rate and body weight changes in neonatal mice (*n* = 5 per group) were observed. Two-day-old neonatal mice were inoculated with PBS (**a**, **b**), MR766 (**c**, **d**), PRVABC59 (**e**, **f**), or DENV-2 (**g**, **h**) strains. Data show the mean ± SD of five mice per group. Statistical analysis *p < 0.05, **p < 0.01, ***p < 0.001

Next, we evaluated the protective effect of Z_EDIII against two ZIKV strains, MR766 and PRVABC59. Pups from immunized parents were challenged with ZIKV strains at a dose of 10^6.8^, 10^6.3^ TCID_50_/mouse and the viral antigenicity was observed in serum at 48 h post-infection. In addition, the survival rates were examined. Despite the level of MR766 in circulation being attenuated by immunization, infected animals failed to survive longer than 10 days. On the other hand, PRVACB59 was not detected when Z_EDIII was administrated and about 60% of infected pups survived (Table 2, Fig. 7a–f). This demonstrates that the Z_EDIII vaccination works as a potential blocker for viral spread.

**Table 2:** Assessment of viremia in neonatal mice serum

Injected group	TCID_50_/ml		
	ZIKV (MR766)	ZIKV (PRVABC59)	DENV-2
PBS	10^2.5^	10^2.3^	10^2.5^
Z_EDIII	10^1.7^	0	10^1.7^

Moreover, we found that Z_EDIII provides cross-protection immunity against a different flavivirus, DENV-type 2. The level of neutralizing antibody against DENV induced by triple vaccination was 20, and the result was comparable against MR766 and PRVABC59 strains (Table 1). The viral detections in neonates from Z_EDIII-immunized and DENV-infected females were lower than in the non-immunized group. The immunization was also beneficial for clinical symptoms and survival. When females were vaccinated with Z_EDIII, their pups were resistant to DENV challenge (Fig. 7g and h).

## Discussion

ZIKV infection can have serious medical consequences, such as Guillain–Barre syndrome, fetal microcephaly, and malformation. Thus, various ZIKV vaccine forms, including purified-inactivated, live-attenuated, and recombinant vaccines have been under development and some of them are under pre-clinical trials [19]. However, no authorized vaccine is available yet, and indeed the current vaccine forms do not fully assure safety and reliability. For example, a subunit vaccine composed of protein fragments can induce toxic immune responses and the pre-existing immunity to viral vector can suppress vaccine efficacy [39]. Moreover, potential tumorigenesis elicited by the insertion of a gene into a DNA or mRNA vaccine [40, 41], as well as unexpected immune response by viral-vectored vaccines [24, 42], can be risk factors. Lately, subunit vaccines utilizing ZIKV EDIII region to provide safety and efficacy has being developed and they protected hosts from ZIKV challenge [16, 21, 43]. An EDIII-encoding subunit vaccine using the *E. coli* expression system is considered as a potent inducer for host immune response with the least side effects [34, 44]. Thus, we evaluated immunogenicity by Z_EDIII immunization and immune responses to ZIKV and DENV infections in immunocompetent mice and their offspring.

The EDIII domain in ZIKV includes a cellular receptor-binding motif and is a potent stimulator of adaptive immunity that stimulates production of neutralizing antibodies and cytokines during infection [13, 16–19]. Z_EDIII immunization promoted humoral response: antigen-specific antibody secretion. We found that Z_EDIII induced protected immunity with a 20-fold increase in neutralizing antibody production when mice were challenged with ZIKV strains (MR766 and PRVABC). Considering that the authorized flavivirus vaccines are to have at least a tenfold boosting effect [39, 45], our vaccine provided partial protective immunity. The lower IgG2c-to-IgG1 ratio by Z_EDIII administration suggests that the cellular immunity is biased to Th2 response. Adjuvants in ZIKV subunit vaccines support neutralizing antibody production [26]. This study used two adjuvants, MPLA and Alum. MPLA is a toll-like receptor 4 (TLR4) inducer that induces a type I response. In contrast, Alum tends to stimulate Th2-oriented response; an established Th2 orientation is not switching to Th1 response [46]. This leads us to assume that repeated Z_EDIII immunizations with Alum adjuvant provides a Th2-dominant condition.

Both vaginal and subcutaneous ZIKV infections induce virus-specific T cell proliferation and the adoptive transfer of the T cells suppresses viral titers during infection. Structural proteins of ZIKV such as E, prM, and C are a major target of CD4+ and CD8+ T cells [19, 47, 48]. IFN-γ production in T cells is critical in protective immunity during ZIKV infection [39, 47, 49–51]. The CD4+ T cell population was the largest by triple immunization and we identified IFN-γ-producing cells. The numbers of such cells was the highest in single-dosed animals and the cell numbers declined as the vaccination was repeated. Production of effector cytokine such as IFN-γ, IL-12, and TNF-α was also stimulated the most by a single dose and the level went down with additional immunization. This is associated with the IgG2c-to-IgG1 ratio; multiple doses induce a Th2-dominant immune response.

Congenital ZIKV transmission is fatal to neonates, and thus a ZIKV vaccine needs to provide protection in fetal and neonatal stages [27, 52, 53]. We immunized females with the Z_EDIII vaccine and examined protection in their neonates. We immunized females with the Z_EDIII vaccine and examined protection against an African (MR766) and ab Asian (PRVABC56) ZIKV strains in neonates [54]. During the infection, antigen specific antibodies were detected in pups and they showed less viremia and increased survival rates. However, the protection was not complete in both African and Asian strain challenged groups. Among 3424 amino acids of ZIKV ORF, 75 to100 amino acid residues showed dissimilarity between the two strains [55]. We speculate that further study is needed to dissect how the genotypes of ZIKV strains regulates immunopathogenesis.

Also, the Z_EDIII induced cross-protection against DENV. The cross-reactivity can induce antibody-dependent enhancement (ADE). This is mediated by the binding of Fcγ receptors and IgG and the structural homology of E protein between ZIKV and other flaviviruses [51, 56, 57]. This similarity provided neutralizing activity for DENV and a protected infected host, but further studies should be conducted to determine whether Z_EDIII induces ADE and protection is acquired from ZIKV-specific neutralizing antibodies.

## Conclusions

This study observed Z_EDIII-mediated humoral immunity that induced antigen-specific IgG and neutralizing antibodies and cellular immunity that promoted effector cytokine production such as IFN-γ, TNF-α, and IL-12. While there was partial protection against ZIKV Asian strain, vaccination induced neutralizing antibodies to another flavivirus, DENV, further manipulations and improvements on the Z_EDIII vaccine platform would be needed to develop a pan-flavivirus vaccine.

## Data Availability

All the data generated during the current study are included in the manuscript.
